# Anti-flotillin-1/2 antibodies in a patient with neurogenic muscle atrophy and mild neuropsychological impairment

**DOI:** 10.1186/s42466-022-00208-6

**Published:** 2022-09-22

**Authors:** Tobias A. Wagner-Altendorf, Klaus-Peter Wandinger, Robert Markewitz, Anna Antufjew, Tobias Boppel, Thomas F. Münte

**Affiliations:** 1 Department of Neurology, University of Lübeck, Lübeck, Germany; 2 Cognitive Neuroscience Lab, Department of Psychology, Northwestern University, 2029 Sheridan Road, Evanston, IL 60208 USA; 3 Institute of Clinical Chemistry, University Hospital Schleswig-Holstein, Lübeck/Kiel, Germany; 4 Department of Neuroradiology, University of Lübeck, Lübeck, Germany

**Keywords:** Anti-flotillin antibodies, Neurogenic muscle atrophy, Limbic encephalitis, Anti-neuronal antibodies, Autoimmune encephalitis

## Abstract

Autoimmune-mediated neural inflammation can affect both the central and the peripheral nervous system. Recently, antibodies against the peripheral membrane protein flotillin have been described in patients with multiple sclerosis, limbic encephalitis and sensorimotor demyelinating polyneuropathy. Here, we report the case of a 75-year-old male patient presenting with slowly progressive muscle weakness, as well as mild cognitive impairment. MR neurography of the leg showed fascicular enlargement and inflammation of ischiadic nerve fibers, while cerebral MRI showed bilateral hippocampal atrophy. Serological testing revealed positive anti-flotillin-1/2 antibodies in serum (1:100) and CSF (1:1). Assuming autoimmune anti-flotillin antibody-associated neurogenic muscle atrophy, the patient was treated with immunoglobulins, which led to a clinical improvement of muscle weakness. In light of the positive anti-flotillin antibodies and the local CNS immunoglobulin production, the mild cognitive impairment and hippocampal atrophy were interpreted as a cerebral involvement in the sense of a subclinical limbic encephalitis. We conclude that anti-flotillin antibodies can be associated with central and peripheral nervous system autoimmunity and should be considered in diagnostical workup.

## Introduction

Autoimmune-mediated neural inflammation can affect both the central and the peripheral nervous system, e.g. in limbic encephalitis and in polyneuropathic syndromes.

Next to well-established anti-neuronal antibodies with intracellular antigens (anti-Hu, Ri, Ma2, GAD, CV2, amphiphysin antibodies) or cell surface antigens (anti-GABAa/b-, NMDA-, AMPA-receptor, LGI1, CASPR2 antibodies), recently antibodies against the cell surface protein flotillin have been described in a patient with limbic encephalitis [11], in a patient with sensorimotor demyelinating polyneuropathy [4], and in patients with multiple sclerosis [7].

Here, we report on a 75-year-old patient presenting with neurogenic muscle atrophy, who showed positive anti-flotillin-1/2 antibodies in serum and CSF.

## Case presentation

A 75-year-old male patient presented with muscle weakness and fatigue, slowly progressive over the last 3–4 years. Over the last 2 years, he had lost 9 kg weight. Additionally, he described suffering from memory impairment.

Clinical examination showed generalized, symmetric, proximally accentuated muscle atrophy with pronounced ubiquitous fasciculations (but no objectifiable paralysis (MRC 5/5)), as well as dysphonia with increased throat clearing. Neuropsychological testing revealed only slight cognitive impairment, but indications of a moderate depression.

**Fig. 1:**
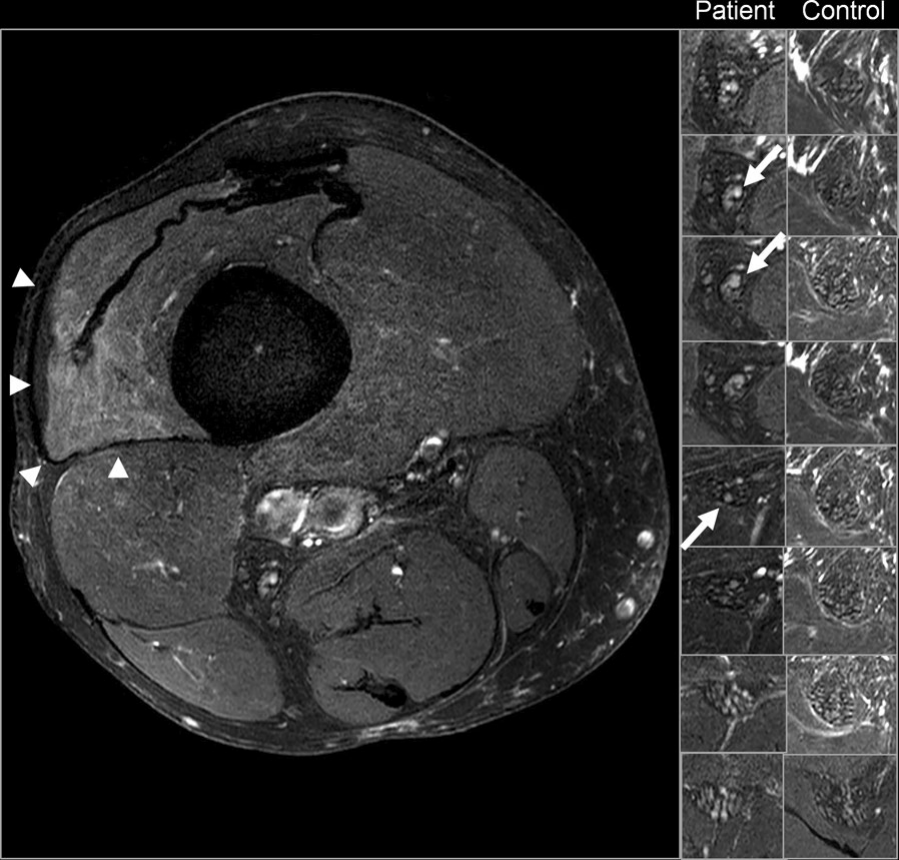
MR neurography (high resolution T2 SPAIR) of the ischiadic nerve showing fascicular enlargement and T2 signal increase of nerve fibers consistent with inflammation (arrows). Denervation edema and a mild atrophy in M. vastus lateralis (arrow heads). Right: Comparison of MR neurography of the patient and MR neurography of an age-matched, healthy control

MR imaging including MR neurography of the leg showed fascicular enlargement and T2 signal increase of ischiadic nerve fibers consistent with inflammation, denervation edema and atrophy in several muscles including M. vastus lateralis and M. adductor magnus (Fig. 1). Laryngoscopy showed atrophic vocal cords with incomplete glottis closure. Electromyography revealed fasciculations in the vastus lateralis muscles, and pathological spontaneous activity in the left gastrocnemius muscle. Motor nerve conduction study of the ulnar and tibial nerves was normal; sensory nerve conduction study revealed slight SNAP (sensory nerve action potential) amplitude reduction in the sural nerves and in the right median nerve, but no reduction in nerve conduction velocity. Repetitive transcranial magnetic stimulation showed extended cortical latency to all extremities.

Serological testing revealed positive IgG antibodies against flotillin-1/2 (1:100), whereas anti-GM1, GM2, GQ1b, IgLON5, Amphiphysin, CASPR2, LGI1, NMDA, AMPA, CV2(CRMP-5), PNMA2(Ma-2/Ta), Ri, Yo, Hu, Recoverin, SOX1, Titin, DPPX antibodies were negative. CSF analysis showed positive IgG antibodies against flotillin-1/2, too (1:1), a normal cell count, an elevated total protein (932 mg/dl (normal range 150–450 mg/dl), local production of IgA (15%) and IgM (50.8%) and negative oligoclonal IgG. ß-Amyloid 1–40, 1–42, Tau, Phospho-Tau were within normal limits.

Cerebral MR imaging revealed hippocampal atrophy and mild global atrophy, as well as several supratentorial T2 hyperintense white matter lesions. EEG showed normal activity in the lower alpha band (8–9 Hz) without epileptiform activity or focal slowing. An extensive neuropsychological examination revealed mild to moderate impairments in verbal memory, working memory, executive attention and non-verbal fluency.

Sonography of the abdomen, coloscopy, MRI of thorax/abdomen, and nephrological and dermatological examination showed no evidence of neoplasia.

The patient was treated with immunoglobulins (30 g/day intravenous for 5 days). On follow-up 4 weeks later, he reported subjective clinical improvement and an overall greater vigor. 8–10 weeks after IVIG therapy, however, the patient reported again a gradual worsening of symptoms. Another IVIG treatment was initiated, which resulted anew in a credible subjective improvement.

## Discussion

We reported on a patient with neurogenic muscle atrophy, showing antibodies against flotillin-1/2 proteins in serum and CSF.

Flotillins are ubiquitously expressed peripheral membrane proteins, involved in axon outgrowth, endocytosis, T cell activation and cell proliferation [12]. As flotillin-1/2 plays a role in trafficking and processing of the amyloid precursor protein [3], (decreased) flotillin serum and CSF levels have been discussed as a biomarker for Alzheimer’s [1, 2].

Anti-flotillin antibodies have recently been described in multiple sclerosis [7], limbic encephalitis [11], sensorimotor demyelinating polyneuropathy [4] as well as in marginal zone lymphoma and paraneoplastic multifocal CNS affection [9]. Anti-flotillin antibodies are described to occur very rarely, if at all, in the normal population; Hahn et al. [7] did not find any anti-flotillin antibodies in 444 healthy blood donors, as well as in 67 patients with other neural autoantibody-associated syndromes.

It has been hypothesized that, as flotillins are implicated in the differentiation of hippocampal neurons [10], anti-flotillin antibodies can impair the functioning of mesial temporal structures and thus present clinically as limbic encephalitis [11]. As flotillins are important for axon regeneration [8], it seems likely to assume that the anti-flotillin antibodies found in our patient are associated with the neurogenic muscle atrophy. This is supported by the observation that clinical improvement could be achieved by immunoglobulins.

Potential differential diagnoses include CIDP (chronic inflammatory demyelinating polyradiculoneuropathy) and FTD-ALS (frontotemporal dementia – amyotrophic lateral sclerosis). While CIDP would be untypical given the proximally accentuated muscle atrophy, the absence of clinical affection of the upper motoneuron, electrophysiologically moderate affection of sensory nerves, signs of inflammation in the MR neurography, and, importantly, the improvement after IVIG therapy speak against ALS.

Extensive tumor search did not reveal any evidence of neoplasia. Still, it may be possible that a tumor that is currently too small to be detected is responsible for the production of the autoantibodies; and paraneoplastic antibody-related diseases are known to possibly precede the diagnosis of the neoplasia for years. Thus, follow-up search for tumor must be considered in the case of initial negative findings.

One case is reported of anti-flotillin antibodies presumably causing paraneoplastic CNS inflammation in a patient with hematological malignancy [9]. Importantly, flotillins are reported to be upregulated in many invasive carcinomas [5].

**Fig. 2:**
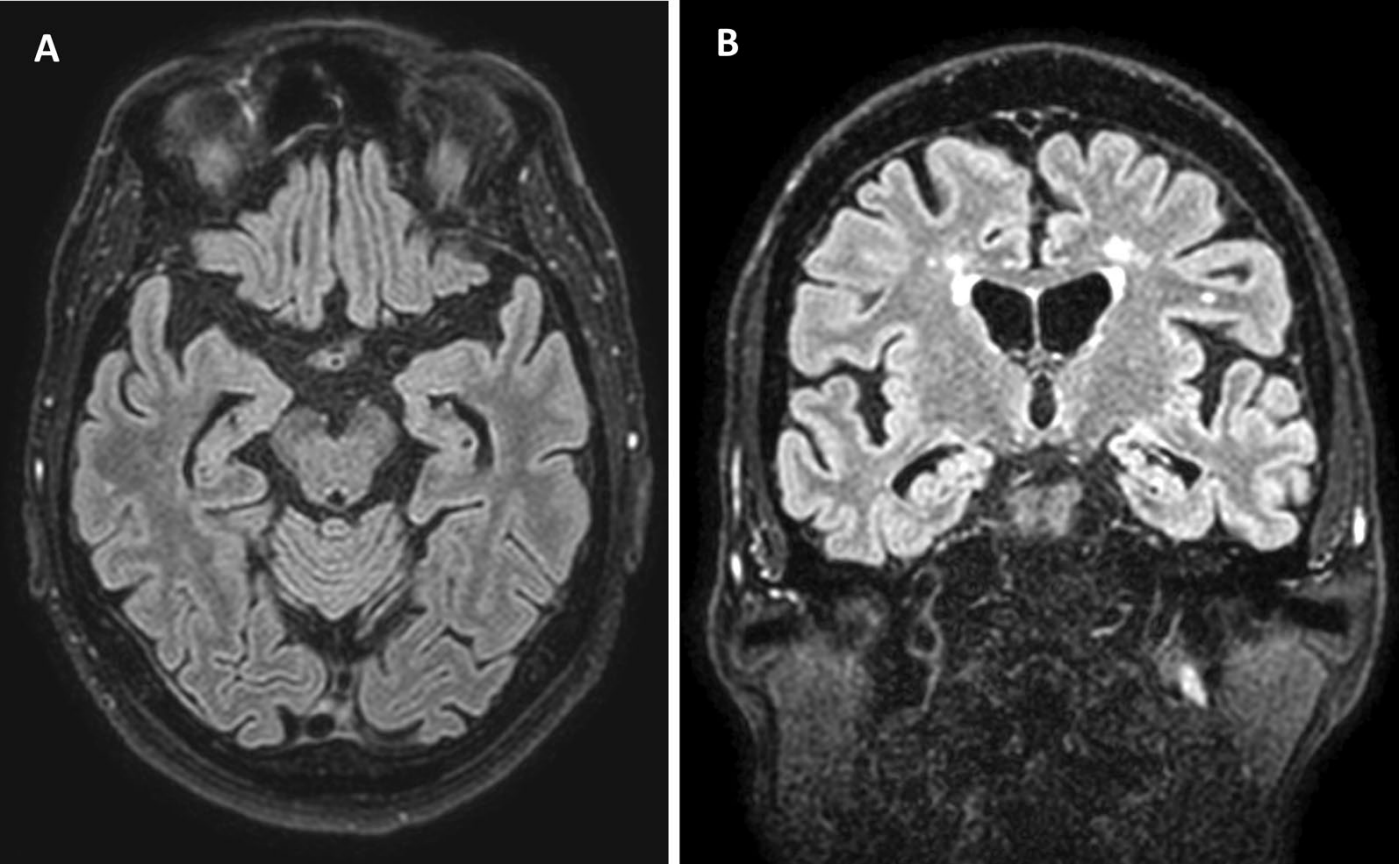
Cerebral MR imaging showing bilateral hippocampal atrophy and mild global atrophy (Fluid attenuated inversion recovery (FLAIR);** A**: axial;** B**: coronar)

The observed slight cognitive impairment presumably corresponds to the hippocampal atrophy (Fig. 2). In light of the positive anti-flotillin antibodies in the CSF, the local immunoglobulin production and one previous report of a limbic encephalitis in conjunction with anti-flotillin antibodies [11], we view this atrophy as likely due to a cerebral involvement in the sense of a subclinical limbic encephalitis.

To conclude, antibodies against the cell surface protein flotillin can be associated with autoimmune affection of both the central and the peripheral nervous system and should be considered in diagnostical workup.

## Data Availability

The raw data supporting the conclusions of this article will be made available by the authors, without undue reservation.
